# 16S rRNA gene sequencing baseline for the atmospheric microbiome of traffic-impacted urban centers in the Klang Valley, Malaysia

**DOI:** 10.1128/mra.00477-26

**Published:** 2026-06-15

**Authors:** Mia Yang Ang, Noor Haziqah Kamaludin, Mohd Talib Latif, Jheng-Jie Jiang, Wan Amir Nizam Wan Ahmad, Norfilza Mohd Mokhtar, Nur Azalina Suzianti Feisal

**Affiliations:** 1 Department of Biomedical Sciences, Jeffrey Cheah Sunway Medical School, Faculty of Medical and Life Sciences, Sunway University 65189, Sunway City, Petaling Jaya, Selangor, Malaysia; 2 Sunway Microbiome Centre, Faculty of Medical and Life Sciences, Sunway University 65189, Sunway City, Petaling Jaya, Selangor, Malaysia; 3 Occupational Health and Safety Risk Management (OHSeRM) Research Initiative Group and Centre for Environmental Health and Safety Study, Faculty of Health Sciences, Universiti Teknologi MARA 54703, Puncak Alam, Selangor, Malaysia; 4 Department of Earth Sciences and Environment, Faculty of Sciences and Technology, Universiti Kebangsaan Malaysia 61775, Bangi, Selangor, Malaysia; 5 Advanced Environmental Ultra Research Laboratory (ADVENTURE) and Department of Environmental Engineering, Chung Yuan Christian University 34900, Taoyuan, Taiwan; 6 Biomedicine Programme, School of Health Sciences, Health Campus, Universiti Sains Malaysia 26689, Kubang Kerian, Kelantan, Malaysia; 7 International Medical School, Management and Science University (MSU) 230176, Shah Alam, Selangor, Malaysia; 8 MSU Centre for Climate Resilience and Strategy (m-CREST), Management and Science University (MSU) 230176, Shah Alam, Selangor, Malaysia; 9 Department of Diagnostic and Allied Health Science, Faculty of Health and Life Sciences, Management and Science University (MSU) 230176, Shah Alam, Selangor, Malaysia; University of Maryland School of Medicine, Baltimore, Maryland, USA

**Keywords:** atmospheric microbiome, urban bioaerosols, 16S rRNA gene sequencing, climate action, sustainable cities

## Abstract

We report the 16S rRNA gene (V3–V4) amplicon dataset of microbial communities from outdoor air filters at three traffic-exposed locations in the Klang Valley, Malaysia. This baseline characterization of the tropical atmospheric microbiome contributes to understanding airborne microbial diversity in Southeast Asian urban environments.

## ANNOUNCEMENT

Urban bioaerosols in tropical regions, such as the Klang Valley, Malaysia, are influenced by warm, humid conditions and traffic-related particulate matter (1, 2). To establish a baseline atmospheric microbiome data set, outdoor air filters were collected from three traffic-exposed primary school sites: Meru (ATM_KC_01_FP07), Damansara (ATM_KC_01_FP09), and Kuala Selangor (ATM_KC_01_FP12).

Sampling was conducted between October and November 2025 using a high-volume air sampling pump. Airborne particulate matter was collected on GF/A glass microfiber filter papers (Whatman, UK; 1.6 µm pore size, 90 mm diameter). At each site, the sampler was placed outdoors within the school environment, and air samples were collected for 12 h, from 7:00 a.m. to 7:00 p.m., at a normal flow rate of 72 L/min. One filter sample was collected per site without field replicates. After sampling, filters were aseptically removed using sterile forceps, placed in sterile containers, transported under cold conditions, and stored at −80°C until DNA extraction. Geographic metadata and sequencing statistics are summarized in Table 1.

**TABLE 1:** Geographic metadata and sequencing statistics for air filter microbiome samples in Klang Valley, Malaysia

Sample ID	Site location	GPS coordinates	Collection date	Raw reads	% Q30	Chao1 index	Shannon (H′)
ATM_KC_01_FP07	Meru, Klang	3.13°N, 101.44°E	21 Oct. 2025	851,798	73.00	260.9	4.93
ATM_KC_01_FP09	Damansara, PJ	3.12°N, 101.62°E	03 Nov. 2025	798,404	74.30	251.08	4.76
ATM_KC_01_FP12	Kuala Selangor	3.34°N, 101.25°E	09 Nov. 2025	903,866	73.10	323.02	5.41

Total genomic DNA was extracted from whole GF/A glass microfiber filter papers using the Qiagen DNeasy PowerSoil Pro Kit (Qiagen, Hilden, Germany), according to the manufacturer’s protocol. DNA concentration and purity were assessed using an Implen NanoQuant Spectrophotometer. The 16S rRNA V3–V4 region was amplified using Illumina-overhang primers: forward primer 5′-TCGTCGGCAGCGTCAGATGTGTATAAGAGACAGCCTACGGGNGGCWGCAG-3′ and reverse primer 5′-GTCTCGTGGGCTCGGAGATGTGTATAAGAGACAGGACTACHVGGGTATCTAATCC-3′.

Libraries were prepared using the Illumina 16S Metagenomic Sequencing Library Preparation protocol with Unique Dual Indexes Set A, purified with AMPure XP beads, pooled, and sequenced on the Illumina MiSeq platform with 300 bp paired-end reads. The tree runs generated 2,554,068 raw paired-end reads, corresponding to approximately 794 Mbp of raw sequence data. Bioinformatics analysis was performed using the QIAGEN CLC Microbial Genomics Module (v23.0), with default trimming and OTU clustering parameters. OTUs were clustered at 97% sequence identity against the Greengenes2 database, release 2024.09 (3).

**Fig 1:**
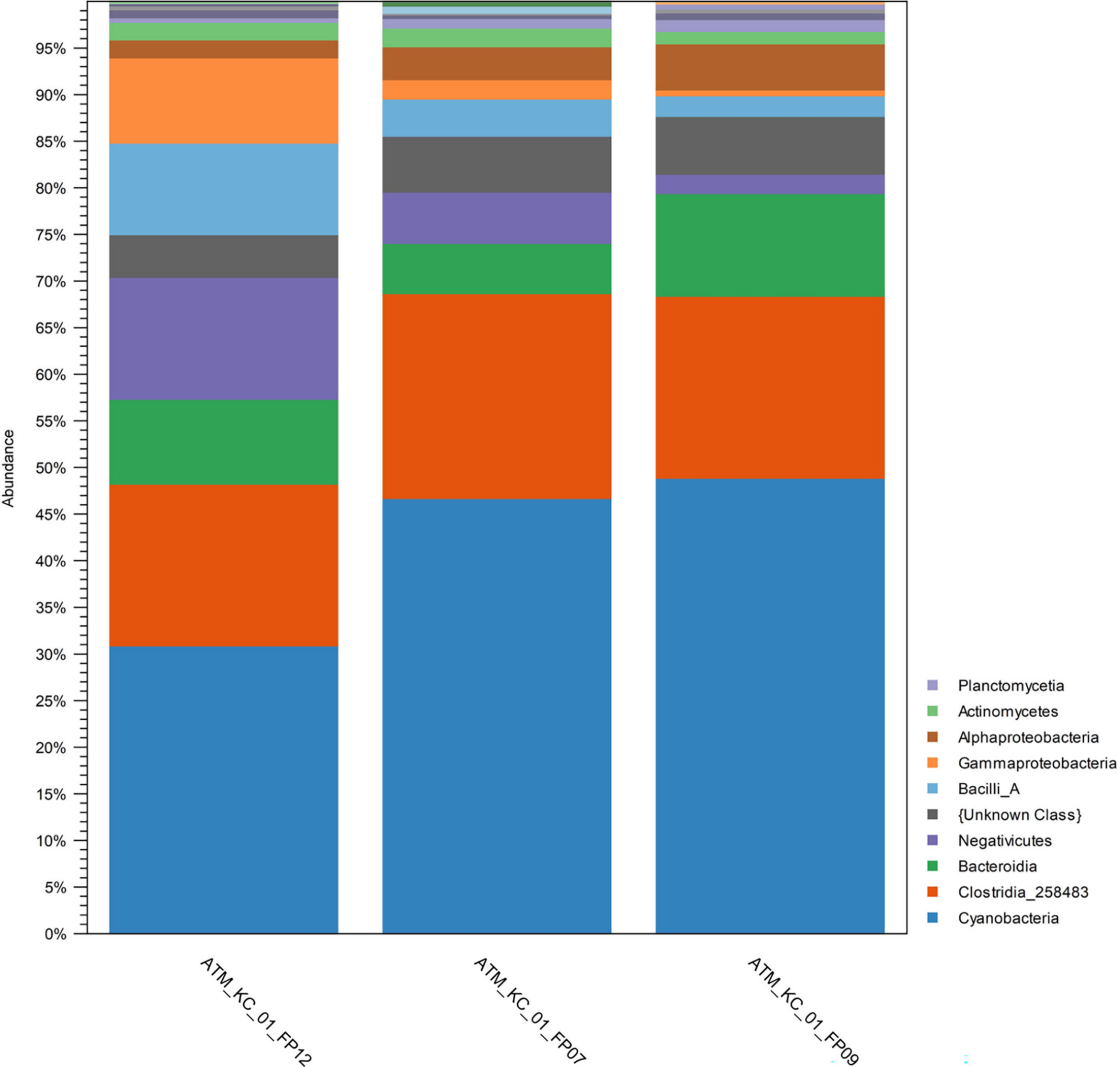
Relative abundance of microbial classes across three Klang Valley sites. The bar chart illustrates the relative abundance of the most prevalent microbial classes across three sampling sites: Meru (ATM_KC_01_FP07), Damansara (ATM_KC_01_FP09), and Kuala Selangor (ATM_KC_01_FP12). Taxonomic assignments were performed at 97% sequence identity against the Greengenes2 database, release 2024.09. Cyanobacteria-related assignments were dominant across the samples, although explicit chloroplast labels were not identified in the current taxonomy output.

Taxonomic profiling identified Cyanobacteria-related assignments as the dominant group across samples (Fig. 1), with higher read counts in ATM_KC_01_FP07 (93,664 reads) and ATM_KC_01_FP09 (91,663 reads) than in ATM_KC_01_FP12 (60,945 reads). Assigned Cyanobacteriota entries included lower-level annotations such as *Caldora* and *Parasynechococcus*, but explicit chloroplast labels were not identified in the current taxonomy output. Thus, any potential contribution from plant-associated or chloroplast-derived DNA requires further validation. Other abundant classes included Clostridia (114,844 total reads), Bacteroidia (49,424 total reads), and Negativicutes (40,768 total reads). Alpha diversity was highest in ATM_KC_01_FP12 and lowest in ATM_KC_01_FP09 (Table 1).

This preliminary data set provides a baseline snapshot of airborne microbial composition across three traffic-exposed Klang Valley locations. Because the study included only three sites, one filter per site, no field replicates, and one comparatively less urbanized site, observed diversity differences should be interpreted cautiously. Future studies with biological replicates, temporal sampling, and expanded site coverage are needed to validate these patterns. This work provides a foundational baseline for future research into urban ecology and environmental health monitoring in Southeast Asian tropical cities (4).

## Data Availability

The raw 16S rRNA gene sequences have been deposited in the NCBI Sequence Read Archive (SRA) under BioProject accession number PRJNA1453751. The individual SRA experiment accessions are SRX32955187 (Meru), SRX32955188 (Damansara), and SRX32955189 (Kuala Selangor).
